# Mindfulness, subjective, and psychological well‐being: A comparative analysis of FFMQ and MAAS measures

**DOI:** 10.1111/aphw.70019

**Published:** 2025-03-17

**Authors:** Anastasia Stuart‐Edwards

**Affiliations:** ^1^ Dhillon School of Business University of Lethbridge Lethbridge AB Canada

**Keywords:** mindfulness, positive psychology, psychological capital, psychological well‐being, subjective well‐being

## Abstract

This study investigates the relationships of mindfulness with subjective well‐being and psychological well‐being through the mediating role of psychological capital. It also compares the Mindful Attention Awareness Scale (MAAS) and the Five Facet Mindfulness Questionnaire (FFMQ) in relation to these outcomes. Using a randomized controlled design, 185 participants from Prolific completed a brief two‐week mindfulness intervention, with one pre‐ and two post‐intervention measures. While the intervention effects were limited, the findings reveal that mindfulness is positively and similarly associated with both SWB and PWB, with stronger indirect links to PWB via PsyCap. While both measures of mindfulness had similar relationships with all outcomes, the multidimensional FFMQ offered additional insights, identifying the “describing” facet as particularly influential for both SWB and PWB, informing new potential paths for theorizing and practice.

## INTRODUCTION

Mindfulness, originating from Eastern meditation traditions, is defined as a cognitive state marked by attention to and awareness of internal and external stimuli, an accepting, nonevaluative attitude, and nonreactivity to inner experiences (Baer et al., 2006). Having permeated Western society, it has garnered strong evidence for its capacity to cultivate various positive characteristics and promote health (Baer & Lykins, 2011; Bishop et al., 2004; Kabat‐Zinn, 2003). Research on mindfulness has primarily targeted its effects on reducing negative outcomes, such as anxiety, depression, and stress, with comparatively less emphasis on its potential to enhance positive outcomes (Baminiwatta & Solangaarachchi, 2021; Stuart‐Edwards et al., 2023). A valuable perspective to investigate these positive outcomes is provided by the discipline of positive psychology, which focuses on studying behaviors, outcomes, and processes that deviate positively, encompassing both individual and collective societal aspects (Seligman, 2002). It encourages a proactive and appreciative view of human potential and capacities, enabling individuals to thrive and flourish (Sheldon & King, 2001). Thus, at their core, both positive psychology and mindfulness research share values and aim to achieve similar goals—namely, to relieve suffering and improve the quality of life (Coo & Salanova, 2018), offering a fertile ground for their integration.

The present study adopts a positive psychology approach to mindfulness, examining its relationship with two facets of well‐being: *subjective well‐being* (SWB, also known as hedonic well‐being) and *psychological well‐being* (PWB, also known as eudaimonic well‐being) (Keyes et al., 2002; Ryan & Deci, 2001). SWB is a global evaluation of affect and life quality (Keyes et al., 2002) consisting of three components – life satisfaction, presence of positive mood, and absence of negative mood, together frequently summarized as happiness (Diener et al., 1999; Ryan & Deci, 2001). PWB reflects optimal human functioning in dimensions such as positive relations, autonomy, environmental mastery, personal growth, purpose in life, and self‐acceptance (Keyes et al., 2002; Ryan & Deci, 2001; Ryff & Singer, 2008). Both aspects are significant, offering complementary approaches to understanding the human pursuit of a fulfilling life (Ryan & Deci, 2001). SWB captures shorter‐term enjoyment and satisfaction derived from positive emotional experiences, while PWB reflects the deeper processes of growth, meaning, and purpose that sustain long‐term fulfillment. Together, they provide a holistic view of well‐being, addressing both immediate happiness and enduring personal development (Ryan & Deci, 2001).

Although various studies have established a link between mindfulness and well‐being, the relationship remains ambiguous due to the frequent conflation of SWB and PWB in these studies, despite their representation of distinct aspects of well‐being (Hanley et al., 2015; Ryan & Deci, 2001). Moreover, the comparative contributions of mindfulness to SWB and PWB have rarely been investigated concurrently. The few studies that have explored both types of well‐being indicate that mindfulness may be more strongly associated with PWB than SWB (Brown & Ryan, 2003; Hanley et al., 2015). The current study draws from two prominent theories—Broaden‐and‐Build theory (Fredrickson, 2001) and Mindfulness‐to‐Meaning theory (Garland et al., 2015)—that focus on subjective and psychological well‐being respectively to theorize its relationship with well‐being. Furthermore, this work examines the role of psychological capital, a core construct in positive psychology, in elucidating the contribution of mindfulness to both aspects of well‐being.

Adding to the complexity, the field also contends with multiple measures to capture mindfulness. Two notable measures are the Five Facet Mindfulness Questionnaire (FFMQ; Baer et al., 2006) and the Mindful Attention Awareness Scale (MAAS; Brown & Ryan, 2003). Both scales aim to capture trait mindfulness, focusing on day‐to‐day emotions, thoughts, and behaviors. However, they differ significantly in two key areas: (1) the dimensionality of the scale, and (2) the specific aspects of mindfulness they encapsulate. The MAAS is a unidimensional scale that characterizes mindfulness as open awareness and ongoing attention to internal and external stimuli (Brown & Ryan, 2003). In contrast, the FFMQ adopts a multidimensional approach, describing mindfulness in terms of five facets: observing, describing, acting with awareness, nonjudging, and nonreacting (Baer et al., 2006, 2008). Previous studies, such as Zhuang et al. (2017), suggest that the MAAS primarily relates to self‐awareness, whereas the FFMQ also implicates emotion regulation and attention control, thus, potentially capturing theoretically distinct mechanisms. With regards to well‐being, studies focusing on SWB often use a unidimensional measure of mindfulness, like MAAS (e.g., Brown et al., 2009; Kong et al., 2014), whereas those investigating PWB tend to use multidimensional scales, such as FFMQ (e.g., Baer et al., 2008, 2012), therefore, it is unclear whether the two scales are differentially related to these two aspects of well‐being or not. Multifaceted scales, such as the FFMQ, can also shed light on more nuanced relationships between various components of mindfulness and well‐being, deepening our understanding of the benefits and mechanisms of mindfulness. Therefore, this study not only examines the relationship between mindfulness and well‐being but also explores whether potentially divergent results are due to the use of different measures of mindfulness.

This research makes three significant contributions. First, it integrates two dominant theories linking mindfulness to different aspects of well‐being, moving the field closer to consensus on the role of mindfulness in positive aspects of well‐being. Unlike a bulk of previous research, this study dynamically tests these relationships as conceptualized by the theories. Second, it compares two commonly used measures of mindfulness, providing clarity on how the specific operationalizations of mindfulness might impact the results. Lastly, employing a randomized controlled design with pre‐test and two post‐test measures, the study highlights an intervention accessible to a broad population due to its low time commitment and no cost. While mindfulness is traditionally induced through established programs such as Mindfulness‐Based Stress Reduction (MBSR) therapy and Mindfulness‐Based Intervention (MBI), their duration and costs pose barriers to widespread adoption. Emerging research suggests that even brief daily practices can lead to structural brain changes associated with regulation (Lutz et al., 2014) and improvements in well‐being (e.g., Berghoff et al., 2017; Grégoire & Lachance, 2015; Hülsheger et al., 2015). This work contributes to the evidence supporting the efficacy of brief online mindfulness interventions to enhance mindfulness.

The next sections present the key ideas of the Broaden‐and‐Build and Mindfulness‐to‐Meaning theories and introduce an integrated approach to this study. This is followed by a description of the study's methodology, the presentation of results, and a discussion of their implications, limitations, and future research directions.

## BROADEN‐AND‐BUILD THEORY

The Broaden‐and‐Build theory (Fredrickson, 2001) posits a virtuous cycle where positive emotions lead to broadened cognitive states and adaptive behavior, which in turn build durable resources for well‐being. Although it did not originally focus on mindfulness, the broaden‐and‐build theory provides an insightful framework on how mindfulness can act to broaden attention by encouraging present‐moment awareness (Good et al., 2016). For instance, Fredrickson et al. (2008) found that participants who practiced loving‐kindness mindfulness meditation reported increased levels of positive emotions, which then led to an increase in mindfulness and well‐being. In a review of the Broaden‐and‐Build theory, Garland et al. (2010) present a body of research that further supports that mindfulness is associated with biophysical changes in brain activity associated with broadened cognition. Mindfulness, therefore, serves as an important “broadening” tool in the context of this theory.

While the Broaden‐and‐Build theory offers valuable insights, it primarily focuses on SWB, specifically positive affect, leaving gaps in our understanding of how mindfulness, as a complex multifaceted trait, contributes to other facets of well‐being. Furthermore, this theory is centered around attention and awareness as the core mechanism without delving into how other aspects of mindfulness—such as describing internal experiences, nonreacting, or nonjudgmental attitude—can further promote various aspects of well‐being. Yet, as mindfulness fosters an attitude of nonjudgment and nonreactivity toward emotional stimuli (Baer et al., 2008), it can serve as a buffer against negative emotions and a promoter of positive emotions (Glomb et al., 2011; Good et al., 2016). By reducing emotional reactivity and facilitating a faster return to an emotional baseline (Teper et al., 2013), mindfulness can help initiate this upward spiral, reinforcing positive emotions (Johnson et al., 2021). Thus, in addition to the attention and awareness emphasized within the Broaden‐and‐Build theory, mindfulness could operate through additional pathways of nonjudgment and nonreactivity. Some of the gaps associated with this framework are resolved within the Mindfulness‐to‐Meaning theory.

## MINDFULNESS‐TO‐MEANING THEORY

The Mindfulness‐to‐Meaning theory (Garland et al., 2015) addresses a gap in the literature by explicitly linking mindfulness to PWB. According to this theory, mindfulness facilitates a cascade of emotional and cognitive regulation changes that promote positive reappraisal which then builds meaning and purpose—critical components of PWB. While Mindfulness‐to‐Meaning theory acknowledges that mindfulness initially prompts decentering and metacognitive awareness—similar to the Broaden‐and‐Build theory—it goes further to assert that mindfulness can also influence the construction of one's self‐narrative that encourages interpreting adverse experiences as opportunities for growth and transformation, thereby contributing to a sense of meaning and purpose in life.

Despite its strengths, the Mindfulness‐to‐Meaning theory is primarily centered on positive reappraisal as the mechanism driving PWB. This focus leaves open questions about how other mechanisms of mindfulness, such as resource‐building, fit into this broader framework. Additionally, it is also unclear whether other facets of mindfulness—describing or nonjudging—play a part in promoting PWB. For instance, the describing facet of FFMQ, which captures how well an individual is able to verbally express their experiences (Baer et al., 2006), has been linked to improved well‐being (e.g., MacDonald & Baxter, 2017; Roemer et al., 2021), yet it is not well‐integrated into existing theoretical perspectives. However, labeling internal and external experiences might play an important role in self‐regulation (Short et al., 2016) which could also assist in the emotional regulation of adverse experiences, thereby, further promoting savoring and a more positive appraisal of those experiences, enhancing PWB.

## THE PRESENT STUDY

The current study builds upon the foundational principles of both the Broaden‐and‐Build theory and the Mindfulness‐to‐Meaning theory, merging them to suggest a step toward an integrated framework. According to this framework, mindfulness enhances resources, thus fostering both.

Drawing from the Broaden‐and‐Build theory (Fredrickson, 2001), the present study posits that mindfulness improves SWB. In support of this theory, various empirical studies exploring the mindfulness‐SWB relationship have reported positive correlations. For example, higher mindfulness levels have been linked with increased positive affect, life satisfaction, and happiness, and reduced negative affect (e.g., Bajaj & Pande, 2016; Choi et al., 2012; Coo & Salanova, 2018; Schutte & Malouff, 2011). However, a recent meta‐analysis reported insufficient evidence to support the positive contribution of mindfulness meditation to happiness as a measure of SWB (Folk & Dunn, 2023), although these findings could be limited due to the lack of multifaceted consideration of mindfulness (Khoury, 2023). Moreover, most studies in this area are cross‐sectional, limiting understanding of the dynamic relationships between variables as theorized by the Broaden‐and‐Build theory. This study extends these findings by examining how different mindfulness measures and facets relate to changes in SWB.

Hypothesis 1 Changes in mindfulness are positively associated with changes in SWB.

Building on the Mindfulness‐to‐Meaning theory (Garland et al., 2015), this study also proposes that mindfulness enhances PWB. Past research has reported positive correlations between mindfulness and multiple indicators of PWB, such as vitality, self‐actualization, autonomy, competence, and relatedness (e.g., Brown & Ryan, 2003; Iani et al., 2017; Stuart‐Edwards, 2023) as well as a composite measure of PWB (e.g., Chang et al., 2015). Additionally, studies have noted increased PWB in individuals practicing mindfulness meditation (Baer et al., 2012; Hanley et al., 2015). However, there is a gap in understanding how different mindfulness facets contribute to PWB and whether dynamic relationships, as posited by the theory, exist. This study aims to address these gaps.

Hypothesis 2 Changes in mindfulness are positively associated with changes in PWB.

The study further integrates insights from the Broaden‐and‐Build theory, suggesting that mindfulness‐induced well‐being gains can be attributed to an increase in personal resources. A specific resource that this paper emphasizes is psychological capital (PsyCap) characterized as an individual's positive psychological state, anchored in four main elements: self‐efficacy, optimism, hope, and resiliency (Luthans et al., 2006). The relevance of PsyCap is evident when considering its profound implications for reduced stress, increased well‐being, more positive attitudes, and superior performance of individuals (Avey et al., 2011; Culbertson et al., 2010; Loghman et al., 2023; Wu & Nguyen, 2019; Youssef‐Morgan & Luthans, 2015), making the identification of factors that enhance PsyCap, like mindfulness, crucial. By choosing PsyCap as a mediator, the study integrates broad mechanisms highlighted in the Broaden‐and‐Build theory (e.g., positive reappraisal), Mindfulness‐to‐Meaning theory (e.g., cognitive reappraisal), and mindfulness research (e.g., emotion regulation, awareness, nonreactivity) into a measurable construct that captures their cumulative effects.

One of the primary routes through which mindfulness can enhance PsyCap is the enhanced self‐regulation of emotions and thoughts. Mindful individuals often exhibit cognitive flexibility and sustained positive affect (Malinowski & Lim, 2015; Roche et al., 2014), allowing them to effectively manage emotional responses in stressful or negative situations. They achieve this by creating a mental distance between themselves and the event, which minimizes emotional reactivity. Simultaneously, they can redirect their cognitive processes towards a more constructive and positive perspective on a situation (Glomb et al., 2011; Good et al., 2016), thereby cultivating PsyCap. Additionally, mindful individuals often approach emotionally challenging situations with a nonreactive attitude, thereby increasing their flexibility and minimizing impulsivity (Good et al., 2016). This could allow them to maintain confidence in the face of challenge, the capability to persevere and maintain a positive outlook, and to bounce back from adversity.

Empirical research, while limited, supports these theoretical claims. For instance, a study by Roche et al. (2014) identified a positive relationship between mindfulness and PsyCap across leaders at varying hierarchical levels. Furthermore, intervention studies have echoed these findings, demonstrating the potency of mindfulness practices in enhancing PsyCap components across various populations, from students to professionals (Phang et al., 2015; Pidgeon et al., 2014; Rinkoff, 2017). Interestingly, certain mindfulness facets, particularly nonreactivity, and nonjudgment, have shown to have strong correlations with PsyCap elements like hope and optimism (Malinowski & Lim, 2015). These findings underscore the potential of mindfulness in positively influencing PsyCap and, by extension, well‐being, prompting a facet‐specific investigation to uncover more nuanced relationships. This study also examines these relationships dynamically, consistent with the Broaden‐and‐Build theory's concept of an upward spiral linking broadened attention, resource‐building, and well‐being.

Hypothesis 3 Changes in mindfulness are positively associated with changes in PsyCap.

Hypothesis 4 Changes in PsyCap mediate the relationship between changes in mindfulness and changes in (a) SWB and (b) PWB.

## METHOD

### Participants and procedure

Participants were 185 individuals located in the United Kingdom, United States, or Canada, and registered on an online survey platform Prolific. The average age was 32 years old (SD = 10 years). Sixty‐seven participants reported to be male (36%), 117 participants reported to be female (64%). One hundred twenty‐five participants reported working full‐time (67%), 47 participants reported working part‐time (25%), and the rest reported not working (8%).

The study utilized a pre‐intervention and two post‐intervention measures design. The study procedure was approved by the University Ethics Board. At Time 1, 201 participants completed the measures followed by a random assignment to either intervention (N = 101) or control (N = 100) condition. In the treatment condition, participants were provided with instructions on how to access free self‐guided mindfulness meditations from the University of California, Los Angeles Mindful Awareness Research Center. They were asked to aim for a minimum of 10–15 minutes per day and encouraged to set a goal in the form of an implementation intention to increase the chances of behavioral compliance (Gollwitzer, 1999). Participants were also provided with a diary to record mindfulness minutes during the two‐week program. In the control group, participants were simply thanked for their participation. At Time 2, participants were invited to complete survey measures again, and 146 participants responded (N = 76 in the intervention group and N = 70 in the control group). The intervention group was also asked to report the number of recorded mindfulness minutes for every day of the two‐week program. The mean total time spent on mindfulness meditation exercises was 95 minutes (SD = 78 min) over the course of two weeks. On average, participants spent 7 minutes (SD = 6 min) per day on mindfulness exercises. At Time 3, 134 participants completed the third and final survey (N = 71 in the intervention group and N = 63 in the control group). The control group was given the same resources for mindfulness intervention as the treatment group for their personal use. Following that, 20 responses in total were deleted due to failing an attention check question, 2 responses were deleted because they had missing data on outcomes, and 2 responses were deleted because they were duplicates. There were 185 participants in the final sample used for analysis.

### Measures

*Mindfulness* was measured using two scales. First, the *Mindful Attention Awareness Scale* (MAAS; Brown & Ryan, 2003) was administered consisting of 15 items. Sample items are “I could be experiencing some emotion and not be conscious of it until some time later” and “I break or spill things because of carelessness, not paying attention, or thinking of something else. Cronbach's alphas ranged from 0.89 to 0.91 across the three surveys. Second, the *Five Facet Mindfulness Questionnaire* (FFMQ; Baer et al., 2006) consisting of 39 items. Sample items are “When I'm walking, I deliberately notice the sensations of my body moving” and “I'm good at finding words to describe my feelings”. The answers for both scales were recorded using a five‐point Likert scale (1 – “Almost never” to 5 – “Almost always”). Cronbach's alpha ranges for this scale were: *FFMQ‐composite* was 0.91 to 0.93, *FFMQ‐OBS* (observing) was 0.80 to 0.85, *FFMQ‐DESC* (describing) was 0.90 to 0.93; *FFMQ‐AWA* (acting with awareness) was 0.91–0.93, *FFMQ‐NJUD* (nonjudgment of inner experiences) was 0.90 to 0.94, and *FFMQ‐NREACT* (nonreactivity to inner experiences) was 0.86 to 0.87.

*PsyCap* was measured using Compound PsyCap Scale (Lorenz et al., 2016) consisting of 12 items. Sample items is “If I should find myself in a jam, I could think of many ways to get out of it”. The answers were recorded using a five‐point Likert scale (1 – “Strongly disagree to 5 – “Strongly agree”). Cronbach's alphas ranged from 0.85 to 0.90 across the three surveys.

*Subjective well‐being* was measured using Lyubomirsky and Lepper's (1999) happiness scale consisting of 4 items. The happiness scale was selected for its brevity and strong psychometric properties, allowing for a reliable assessment of overall SWB. Its convergence with other SWB measures supports its use in capturing this construct (Diener et al., 2010; Lyubomirsky & Lepper, 1999). Sample items are “In general, I consider myself …” and “Compared to most of my peers, I consider myself …”. The answers were recorded using a five‐point Likert scale (1 – “Not a very happy person” to 5 – “A very happy person”). Cronbach's alphas ranged from 0.85 to 0.92 across the three surveys.

*Psychological well‐being* was measured using Diener et al.’s (2010) scale consisting of 8 items (e.g., “I lead a purposeful and meaningful life”). Sample items are “I lead a purposeful and meaningful life” and “My social relationships are supportive and rewarding”. The answers were recorded using a five‐point Likert scale (1 – “Strongly disagree to 5 – “Strongly agree”). Cronbach's alphas were 0.90 in all three surveys.

### Analysis

To test hypothesized relationships, the approach developed by Chen et al. (2011) was employed. This approach involves two key steps: estimating change over time in each variable and then assessing the relationships between these changes. Each slope represents the change in scores over time, with positive values indicating growth and negative values indicating a reduction. Because these slopes can vary across individuals, they serve as an index of individual change in the respective variables and can be included in ordinary least square (OLS) regression models (Chen et al., 2011).

In the first step, temporal changes were assessed with mixed‐effects growth models following the guidelines by Bliese and Ployhart (2002) and using R package “nlme” (Pinheiro et al., 2021). The intraclass correlation coefficients (ICC1) for the various scales ranged from .67 to .87, indicating a substantial portion of nonindependence between observations, thus justifying the use of mixed‐effects modeling. Significant time effects were observed for *FFMQ‐composite* (*B* = 0.07, *SE* = 0.02, *p* < .001), *FFMQ‐OBS* (*B* = 0.04, *SE* = 0.02, *p* < .05), *FFMQ‐DESC* (*B* = 0.08, *SE* = 0.03, *p* < .01), *FFMQ‐AWA* (*B* = 0.07, *SE* = 0.02, *p* < .01), *FFMQ‐NREACT* (*B* = 0.09, *SE* = 0.02, *p* < .001), *PsyCap* (*B* = 0.05, *SE* = 0.02, *p* < .05), *SWB* (*B* = 0.07, *SE* = 0.02, *p* < .001), and *PWB* (*B* = −0.07, *SE* = 0.02, *p* < .01), indicating an average positive or negative change over time. The effect was marginally significant for *FFMQ‐NJUD* (*B* = 0.06, *SE* = 0.03, *p* = .051) and not significant for *MAAS* (*B* = 0.02, *SE* = 0.02, *p* = .392), but slopes were still calculated for these variables to provide a comprehensive view of the dynamics of all variables involved, regardless of their individual significance levels. Before calculating slopes, the error structures of each variable were assessed in terms of autocorrelation errors (where responses closer in time are more strongly related than those farther apart) and heteroscedasticity errors (where variability in responses changes over time). While no evidence of autocorrelation was found, heteroscedasticity was observed for *FFMQ‐composite*, *FFMQ‐DESC*, *PsyCap*, and *PWB*, so the calculation of slopes was controlled for heteroscedasticity for these variables.

In the second step, OLS regression was used to test the hypothesized relationships. Specifically, changes in the dependent variable, represented by within‐group slopes, were regressed on changes in the independent variable, also represented by within‐group slopes. This was done while controlling for the initial value of the dependent variable at Time 1 and the average value over of the independent variable over the time period from Time 1 to Time 3 (Chen et al., 2011). Missing data was handled using pairwise deletion to maximize sample size. The analyses were also conducted controlling for age and gender; however, as these variables were not significant, they were not included in the final analyses reported in the manuscript.

Mediation effects were calculated using mediation analysis with 5,000 bootstrap samples in the R package “mediation” (Tingley et al., 2014). Missing data were handled using listwise deletion as mediation effects can only be estimated on models with an equal number of observations.

## RESULTS

### Correlations

Mindfulness, as measured by FFMQ and MAAS, correlated positively with both SWB (*r*_FFMQ_ = .60, *p* < .05; *r*_MAAS_ = .47, *p* < .05) and PWB (*r*_FFMQ_ = .54, *p* < .05; *r*_MAAS_ = .40, *p* < .05) as well as with PsyCap (*r*_FFMQ_ = .61, *p* < .05; *r*_MAAS_ = .38, *p* < .05). Mindfulness facets exhibited different patterns of correlations with the outcomes (see Table S1). All correlations are in predicted directions.

### Intervention effects

**FIGURE 1:**
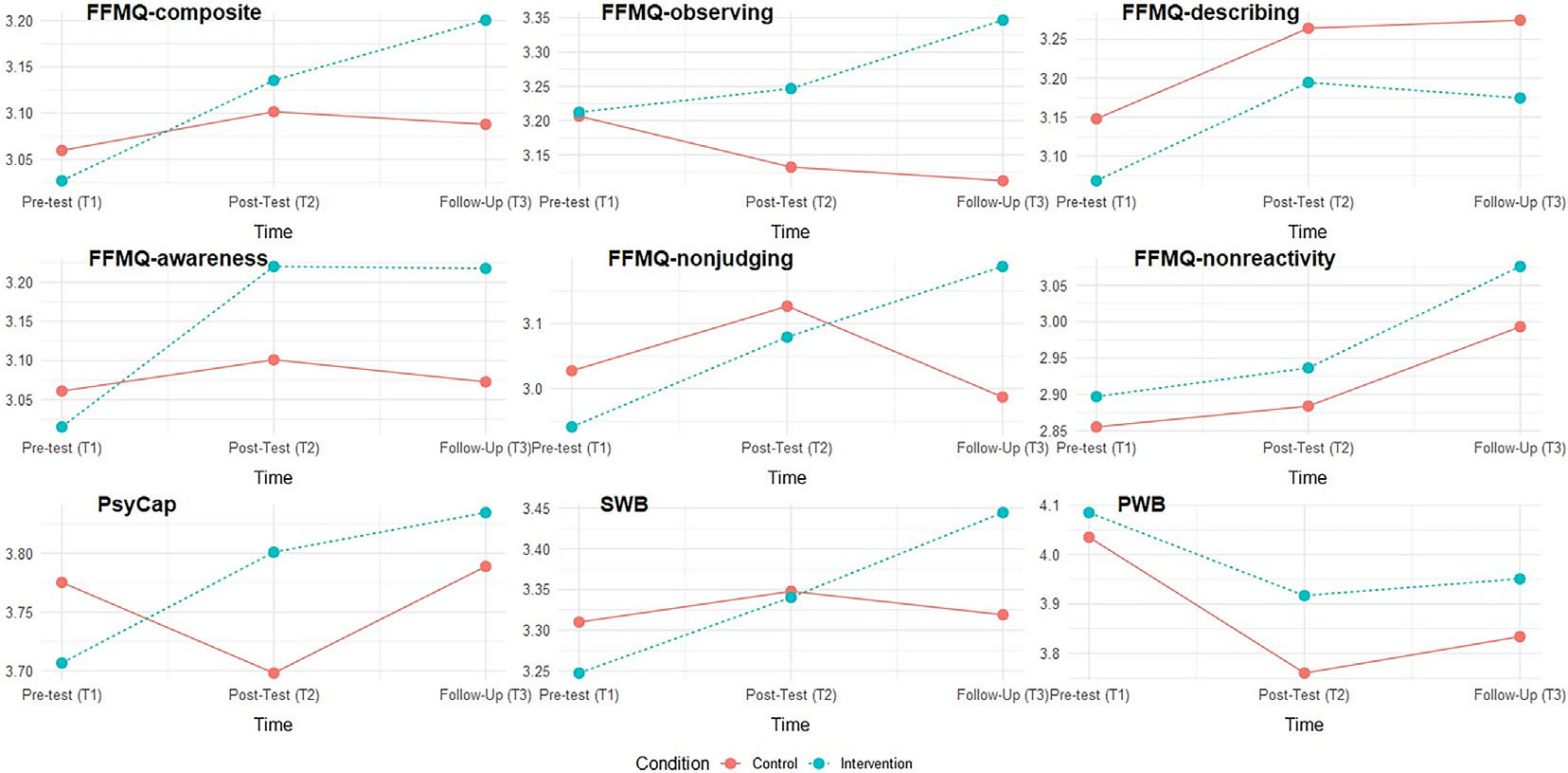
Changes in mean scores of mindfulness, PsyCap, SWB, and PWB across three time points.

Table 1 presents the mean scores and standard deviations for each variable, along with the effect sizes and p‐values for both groups. The intervention group showed a small increase in *FFMQ‐composite* from pre‐training to follow‐up (*d* = .34), which was significantly different from the control group (*p* = .010) where only a negligible increase from pre‐training to follow‐up was observed (*d* = .08). A small increase was seen in the *FFMQ‐OBS* in the intervention group from pre‐training to follow‐up (*d* = .20), which differed significantly from the control group (*p* = .017) where a small decrease (*d* = −.11) was recorded. Both the intervention and control groups showed a small increase in the *FFMQ‐DESC* from pre‐training to follow‐up (*d* = .12 and *d* = .15 respectively); however, the difference between groups was not statistically significant (*p* = .208). The intervention group showed a moderate increase in the *FFMQ‐AWA* (*d* = .27), whereas the control group showed a negligible increase (*d* = .06) but the difference between groups was not statistically significant (*p* =. 306). The intervention group showed a small increase in the *FFMQ‐NJUD* (*d* = .27), while the control group showed a negligible decrease (*d* = −.03); this difference was significant (*p* = .022). A small to moderate increase was noted in the *FFMQ‐NREACT* in both the intervention and control groups from pre‐training to follow‐up (*d* = .24 and *d* = .19 respectively) which was not a significant difference (*p* = .368). There was also a small increase in *MAAS* in the intervention group (*d* = .22) while the control group experienced a negligible decrease (*d* = −.05), but the difference was not statistically significant (*p* = .050). The intervention group showed a small increase in *PsyCap* (*d* = .21) in the intervention group and a negligible increase in the control group (*d* = .01), but the difference was not statistically significant (*p* = .086). A small increase in *SWB* was observed in the intervention group (*d* = .22), while the control group showed a negligible change (*d* = .01); however, the groups were not statistically different (*p* = .167). Both intervention and control groups showed a small decrease in *PWB* from pre‐training to follow‐up (*d* = −.15 and *d* = −.23 respectively), but the difference was not significant (*p* = .623). The changes in these variables are visualized in Figure 1.

**TABLE 1:** Within‐group means, standard deviations, effect sizes, and ANOVA results for intervention and control groups.

	Intervention group					Control group					*p*
	Mean (SD)			*d*_*1*_	*d*_*2*_	Mean (SD)			*d*_*1*_	*d*_*2*_	
	Pre‐training	Post‐training	Follow‐up			Pre‐training	Post‐training	Follow‐up			
FFMQ‐composite	3.03 (0.48)	3.13 (0.48)	3.20 (0.55)	.22	.34	3.04 (0.55)	3.10 (0.59)	3.08 (0.63)	.11	.08	.010**
FFMQ‐OBS	3.26 (0.65)	3.26 (0.67)	3.35 (0.69)	.08	.20	3.20 (0.71)	3.13 (0.73)	3.11 (0.75)	−.09	−.11	.017*
FFMQ‐DESC	3.07 (0.81)	3.22 (0.85)	3.18 (0.93)	.18	.12	3.14 (0.83)	3.26 (0.88)	3.27 (0.86)	.15	.15	.208
FFMQ‐AWA	3.01 (0.77)	3.19 (0.78)	3.22 (0.79)	.23	.27	3.00 (0.91)	3.10 (0.84)	3.06 (0.91)	.11	.06	.306
FFMQ‐NREACT	2.90 (0.73)	2.93 (0.62)	3.08 (0.72)	.04	.24	2.86 (0.72)	2.88 (0.70)	2.99 (0.73)	.04	.19	.368
FFMQ‐NJUD	2.94 (0.90)	3.07 (0.84)	3.19 (0.92)	.15	.27	2.99 (0.84)	3.13 (0.86)	2.97 (0.93)	.16	−.03	.022*
MAAS	3.17 (0.59)	3.25 (0.58)	3.30 (0.56)	.13	.22	3.17 (0.68)	3.23 (0.65)	3.14 (0.74)	.09	−.05	.050
PsyCap	3.71 (0.59)	3.80 (0.66)	3.84 (0.63)	.15	.21	3.78 (0.60)	3.70 (0.71)	3.79 (0.59)	−.13	.01	.086
SWB	3.25 (0.89)	3.33 (1.02)	3.45 (1.01)	.08	.22	3.32 (0.90)	3.35 (1.01)	3.33 (0.98)	.03	.01	.167
PWB	4.08 (0.84)	3.89 (0.66)	3.96 (0.75)	−.25	−.15	4.03 (0.90)	3.76 (0.81)	3.84 (0.78)	−.33	−.23	.623

*Notes*. FFMQ = Five Facet Mindfulness Questionnaire. FFMQ‐OBS = observing. FFMQ‐DESC = describing. FFMQ‐AWA = acting with awareness. FFMQ‐NJUD = nonjudgment of inner experiences. FFMQ‐NREACT = nonreactivity. MAAS = Mindful Attention Awareness Questionnaire. PsyCap = psychological capital. SWB = subjective well‐being. PWB = Psychological well‐being. The *d* column shows Cohen's d effect sizes between pre‐training and follow‐up: d_1_ is pre‐intervention to post‐intervention, d_2_ is pre‐intervention to follow‐up. Cohen's *d* = .3 is small, .5 is medium, .8 is large. The *p* column shows *p*‐values from condition ✕ time interaction from repeated measures ANOVA.

* p < .05,

** p < .01.

### Hypothesis testing

Hypothesis 1 predicted a positive relationship between changes in mindfulness and changes in SWB. The results reported in Table 2 support this hypothesis. Mindfulness change was positively associated with a change in *SWB* as measured by *FFMQ‐composite* (β = .36, *SE* = .07, *p* < .001) and *MAAS* (β = .25, *SE* = .06, *p* < .001). Adding changes in these measures of mindfulness explained an additional 9% and 6% of the change in *SWB* correspondingly. *FFMQ‐composite* had a slightly stronger association with *SWB* than *MAAS* but the effect sizes were comparable. By‐facet analysis of FFMQ revealed that only changes in *FFMQ‐DESC* (β = .29, *SE* = .12, *p* < .05) were associated with changes in *SWB* explaining additional 11% of variance in the changes of this outcome.

**TABLE 2:** Result from linear regression for SWB.

*Predictors*	Model 1		Model 2		Model 3		Model 4		Model 5		Model 6	
	*B*	*SE*	*B*	*SE*	*B*	*SE*	*B*	*SE*	*B*	*SE*	*B*	*SE*
(intercept)	.00	.06	.00	.06	.00	.06	.00	.06	.00	.06	.00	.06
SWB time 1	.36***	.08	.37***	.07	.40***	.08	.42***	.07	.46***	.07	.42***	.07
FFMQ‐composite (average)	.24**	.08	.02	.08								
FFMQ‐composite (change)			.36***	.07								
FFMQ‐OBS (average)					.15*	.07	.00	.10				
FFMQ‐DESC (average)					.13	.08	−.09	.12				
FFMQ‐AWA (average)					.04	.08	−.04	.09				
FFMQ‐NJUD (average)					−.06	.08	−.09	.08				
FFMQ‐NREACT (average)					.07	.08	.04	.07				
FFMQ‐OBS (change)							.14	.10				
FFMQ‐DESC (change)							.29*	.12				
FFMQ‐AWA (change)							.13	.09				
FFMQ‐NJUD (change)							.02	.07				
FFMQ‐NREACT (change)							.11	.07				
MAAS (average)									.08	.07	.07	.07
MAAS (change)											.25***	.06
R^2^	0.28		0.37		0.31		0.42		0.25		0.31	
ΔR^2^			.09***				.11***				.06***	

*Notes*. N = 185. B = standardized regression coefficient. SE = standard error. SWB = subjective well‐being. FFMQ = Five Facet Mindfulness Questionnaire. FFMQ‐OBS = observing. FFMQ‐DESC = describing. FFMQ‐AWA = acting with awareness. FFMQ‐NJUD = nonjudgment of inner experiences. FFMQ‐NREACT = nonreactivity. MAAS = Mindful Attention Awareness Questionnaire.

* *p* < .05,

** *p* < .01,

*** *p* < .001.

Hypothesis 2 predicted a positive relationship between changes in mindfulness and changes in PWB. The results are presented in Table 3. Mindfulness change was positively associated with a change in *PWB* as measured by *FFMQ‐composite* (β = .42, *SE* = .08, *p* < .001) and *MAAS* (β = .39, *SE* = .07, *p* < .001) explaining additional 12% and 15% of variance in *PWB* change respectively. *FFMQ‐composite* had a slightly stronger association with *PWB* than *MAAS,* but the effect sizes were similar. These results support Hypothesis 2. By‐facet analysis of FFMQ revealed that changes in *FFMQ‐DESC* (β = .40, *SE* = .12, *p* < .01), *FFMQ‐AWA* (β = .19, *SE* = .09, *p* < .05), and *FFMQ‐NREACT* (β = .18, *SE* = .07, *p* < .05) were associated with changes in PWB. Collectively, mindfulness facets explained additional 17% of variance in the outcome.

**TABLE 3:** Result from linear regression for PWB.

*Predictors*	Model 1		Model 2		Model 3		Model 4		Model 5		Model 6	
	*B*	*SE*	*B*	*SE*	*B*	*SE*	*B*	*SE*	*B*	*SE*	*B*	*SE*
(intercept)	.00	.07	.00	.07	.00	.07	.00	.06	.00	.07	.00	.07
PWB time 1	−.34***	.08	−.31***	.08	−.31***	.08	−.24**	.08	−.20*	.08	−.23**	.07
FFMQ‐composite (average)	.29***	.08	.04	.09								
FFMQ‐composite (change)			.42***	.08								
FFMQ‐OBS (average)					.18*	.07	.19	.11				
FFMQ‐DESC (average)					.26**	.09	−.09	.13				
FFMQ‐AWA (average)					−.16	.09	−.29**	.10				
FFMQ‐NJUD (average)					.11	.09	.12	.08				
FFMQ‐NREACT (average)					.06	.08	.06	.08				
FFMQ‐OBS (change)							−.09	.11				
FFMQ‐DESC (change)							.40**	.12				
FFMQ‐AWA (change)							.19*	.09				
FFMQ‐NJUD (change)							−.02	.07				
FFMQ‐NREACT (change)							.18*	.07				
MAAS (average)									.06	.08	.03	.07
MAAS (change)											.39***	.07
R^2^	.09		.21		.16		.33		.04		.19	
ΔR^2^			.12***				.17***				.15***	

*Notes*. N = 184–185. B = standardized regression coefficient. SE = standard error. PWB = psychological well‐being. FFMQ = Five Facet Mindfulness Questionnaire. FFMQ‐OBS = observing. FFMQ‐DESC = describing. FFMQ‐AWA = acting with awareness. FFMQ‐NJUD = nonjudgment of inner experiences. FFMQ‐NREACT = nonreactivity. MAAS = Mindful Attention Awareness Questionnaire.

* *p* < .05,

** *p* < .01,

*** *p* < .001.

Hypothesis 3 predicted a positive relationship between changes in mindfulness and changes in PsyCap. The results are summarized in Table 4. Mindfulness change was positively associated with a change in *PsyCap* as measured by *FFMQ‐composite* (β = .28, *SE* = .05, *p* < .001) and *MAAS* (β = .25, *SE* = .04, *p* < .001) which explained additional 5% and 6% of variance in *PsyCap* change respectively. The effects sizes were nearly identical. Hypothesis 3 was supported. By‐facet analysis of FFMQ revealed that only changes in *FFMQ‐AWA* (β = .14, *SE* = .06, *p* < .05) and *FFMQ‐NREACT* (β = .21, *SE* = .05, *p* < .001) were associated with changes in PsyCap, explaining additional 9% of variance in the outcome.

**TABLE 4:** Result from linear regression for PsyCap.

*Predictors*	Model 1		Model 2		Model 3		Model 4		Model 5		Model 6	
	*B*	*SE*	*B*	*SE*	*B*	*SE*	*B*	*SE*	*B*	*SE*	*B*	*SE*
(intercept)	.00	.04	.00	.04	.00	.04	.00	.04	.00	.05	.00	.04
PsyCap time 1	.64***	.05	.69***	.05	.64***	.05	.69***	.05	.73***	.05	.74***	.04
FFMQ‐composite (average)	.27***	.05	.10	.06								
FFMQ‐composite (change)			.28***	.05								
FFMQ‐OBS (average)					.10*	.05	.05	.06				
FFMQ‐DESC (average)					.07	.06	.03	.08				
FFMQ‐AWA (average)					.05	.06	−.01	.06				
FFMQ‐NJUD (average)					.11*	.06	.07	.05				
FFMQ‐NREACT (average)					.09	.05	.09	.05				
FFMQ‐OBS (change)							−.01	.06				
FFMQ‐DESC (change)							.01	.08				
FFMQ‐AWA (change)							.14*	.06				
FFMQ‐NJUD (change)							.09	.04				
FFMQ‐NREACT (change)							.21***	.05				
MAAS (average)									.16**	.05	.13**	.04
MAAS (change)											.25***	.04
R^2^	.66		.71		.66		.75		.63		.69	
ΔR^2^			.05***				.09***				.06***	

*Notes*. N = 181–182. B = standardized regression coefficient. SE = standard error. PsyCap = psychological capital. FFMQ = Five Facet Mindfulness Questionnaire. FFMQ‐OBS = observing. FFMQ‐DESC = describing. FFMQ‐AWA = acting with awareness. FFMQ‐NJUD = nonjudgment of inner experiences. FFMQ‐NREACT = nonreactivity. MAAS = Mindful Attention Awareness Questionnaire.

* *p* < .05,

** *p* < .01,

*** *p* < .001.

Hypothesis 4 predicted that changes in PsyCap will mediate the relationship between changes in mindfulness and changes in both well‐being indicators. For *SWB*, mediation effects through PsyCap were significant for *MAAS* (β = .10, 95% CI [.02; .20], *p* < .05) but not for *FFMQ*. For *PWB*, PsyCap mediated relationships with *FFMQ‐composite* (β = .17, 95% CI [.10; .24], *p* < .001) and *MAAS* (β = .14, 95% CI [.09; .21], *p* < .001). By‐facet analysis of FFMQ revealed that PsyCap also mediated the relationships between *FFMQ‐AWA* (β = .11, 95% CI [.02; .21], *p* < .05) and *FFMQ‐NREACT* (β = .08, 95% CI [.04; .22], *p* < .001). Full results can be found in Table S2.

## DISCUSSION

This paper aimed to examine the role of mindfulness in enhancing subjective and psychological well‐being by building psychological resources—based on an integration of Broaden‐and‐Build (Fredrickson, 2001) and Mindfulness‐to‐Meaning (Garland et al., 2015) theories. Moreover, this study compared the FFMQ and MAAS mindfulness measures, assessing their associations with well‐being outcomes diverge or converge. Employing a randomized controlled brief online mindfulness intervention with pre‐test and two post‐test measures, the findings confirm that mindfulness improves both subjective and psychological well‐being. The results demonstrate that both the FFMQ‐composite and the MAAS significantly predicted PWB and SWB, with similar direct effect sizes. However, while the overall predictive strength was similar, the FFMQ's multidimensional framework offers additional insights by highlighting specific facets of mindfulness, such as describing and nonreactivity, which may contribute to particular aspects of well‐being. The mediation analyses revealed that both FFMQ‐composite and MAAS have indirect effects on SWB and PWB through PsyCap, with slightly stronger mediation effects observed for PWB, supporting Mindfulness‐to‐Meaning theory and offering a path to its integration with the Broaden‐and‐Build theory.

Empirical evidence for the relationship between mindfulness and well‐being has been complicated by the diverse conceptualizations and measures of both constructs, challenging the generalizability of findings (Hanley et al., 2015). This study addressed this issue by categorizing well‐being into subjective and psychological domains and employing two widely used mindfulness measures, FFMQ and MAAS. While small differences were observed in the strengths of association between mindfulness (measured as a composite FFMQ and MAAS) and both aspects of well‐being, the direct relationships were notably similar for SWB and PWB. This aligns with previous studies examining both measures of well‐being concurrently (Brown & Ryan, 2003; Hanley et al., 2015). Furthermore, these findings support both the Broaden‐and‐Build theory (Fredrickson, 2001), which emphasizes SWB, and the Mindfulness‐to‐Meaning theory (Garland et al., 2015), which emphasizes PWB. Together, these results suggest that mindfulness has the potential to enhance both happiness and deeper psychological functioning, underscoring its value as a tool for promoting holistic well‐being.

Furthermore, the use of two measures of mindfulness (FFMQ and MAAS) allowed for a more comprehensive examination of these relationships, demonstrating that while the measures capture different facets of mindfulness, they converge in their overall association with well‐being. Theoretically, this convergence suggests that mindfulness, as a construct, is robust across different measurement approaches, whether captured through the multidimensional FFMQ or the unidimensional MAAS. Practically, these findings highlight the flexibility of measurement tools in mindfulness research and practice, particularly in understanding its impact on well‐being. For situations where simplicity and brevity are necessary, MAAS can serve as an effective measure of mindfulness. In contrast, the FFMQ's multidimensional nature offers the advantage of identifying specific facets of mindfulness that may be more relevant for certain outcomes, as discussed later.

Interestingly, despite the Broaden‐and‐Build theory positing that enhanced cognition facilitates resource building, changes in PsyCap—a psychological resource—were not only linked to mindfulness and PWB but also mediated this relationship more robustly than for SWB. PsyCap, which encompasses hope, resilience, efficacy, and optimism (Luthans et al., 2006), aligns closely with the eudaimonic aspects of PWB, such as personal growth and purpose in life. Its stronger mediation effect for PWB suggests that mindfulness's capacity to build psychological resources is particularly relevant for supporting deeper psychological functioning and long‐term well‐being, as emphasized in the Mindfulness‐to‐Meaning theory. In contrast, the comparatively weaker mediation effect for SWB indicates that while the hedonic aspects of well‐being, such as happiness, are likely influenced more directly by immediate emotional processes (e.g., positive affect), resource accumulation also contributes to SWB and should not be overlooked. This finding highlights the complementarity of the Broaden‐and‐Build theory and the Mindfulness‐to‐Meaning theory in explaining how mindfulness relates to well‐being. While the former focuses on the broadening of positive emotions to foster immediate happiness and satisfaction (SWB), the latter emphasizes the deeper meaning‐making and resource‐building processes essential for psychological flourishing (PWB). Together, these theories describe distinct yet interconnected pathways through which mindfulness influences well‐being. Broader frameworks, such as Positive Psychology (Seligman, 2002) and Conservation of Resources theory (Hobfoll, 1989), may help integrate these perspectives. For example, positive psychology links mindfulness to enhancing positive experiences and fulfillment (e.g., Ivtzan et al., 2016), while Conservation of Resources theory frames mindfulness as a means of building and protecting psychological resources necessary for a broad range of well‐being outcomes (e.g., Zivnuska et al., 2016). This finding calls for an integrated theoretical framework that synthesizes elements of the Broaden‐and‐Build theory and the Mindfulness‐to‐Meaning theory. Such a framework would capture the dual pathways through which mindfulness influences well‐being: one pathway emphasizing the cultivation of immediate positive emotions and expanded cognitive processing that directly enhances SWB, and another focusing on the gradual accumulation of psychological resources and meaning‐making processes that contribute more robustly to PWB. These pathways are not mutually exclusive but rather are complimentary and overlapping, offering a comprehensive view of how mindfulness operates across different dimensions of well‐being.

A key contribution of this study is the dissection of mindfulness facets and their impact on outcomes. While both the Broaden‐and‐Build and Mindfulness‐to‐Meaning theories predict outcomes based on the awareness and attention components, the results of the present study highlight the “describing” facet of mindfulness as pivotal for both outcomes—yet overlooked in theoretical models. This is consistent with several previous studies that have also identified “describing” as a significant contributor to psychological well‐being (MacDonald & Baxter, 2017; Roemer et al., 2021). “Describing” involves verbally expressing one's experiences (Baer et al., 2006), akin to affect labeling, which research shows dampens emotional intensity (Lieberman et al., 2007) and fosters emotion regulation (Marks et al., 2019). Describing, therefore, could interrupt a cycle of self‐critical or harsh thoughts preventing individuals from becoming consumed by negative thoughts (Pepping et al., 2013), thereby promoting both SWB and PWB. Practically, this could inform targeted interventions that specifically emphasize verbalization of emotions and thoughts, such as Dialectical Behavior Therapy (Linehan, 1993), to promote different aspects of well‐being.

The intervention in this study required a minimal time commitment, with participants asked to aim for 10–15 minutes per day, though the average time reported was six minutes. However, its effectiveness was limited. One reason for this may be that the brief duration (two weeks) and low intensity (10–15 minutes per day) were insufficient to produce a meaningful shift in mindfulness. Another reason could be the existing but limited malleability of trait mindfulness. Research has shown that mindfulness can be cultivated through deliberate practice, even for trait‐level measures (Kiken et al., 2015; Quaglia et al., 2016). However, short‐term interventions, such as the one used in this study, may be insufficient to produce significant shifts in trait mindfulness. Evidence suggests that the length of individual mindfulness sessions or type of meditation may be less crucial than the overall length of lifetime practice for mindfulness development (Carmody & Baer, 2009; Soler et al., 2014). Consequently, longer interventions may be necessary to foster substantial development across all facets of mindfulness. The third possible explanation is that mindfulness facets do not develop uniformly over time. For instance, following an MBSR course, initial improvements were noted in decentering and nonreactivity, with later changes observed in observing, acting with awareness, and nonjudging (Davis et al., 2024). It is plausible that the intervention used here promoted quicker changes in facets such as observing and nonjudging, while other facets would evolve later. Given that shifts in these facets are essential for the intervention's impact on outcomes (e.g., Haenen et al., 2016; Kinnunen et al., 2020), modest changes in mindfulness facets may explain the absence of immediate improvements in PsyCap, SWB, and PWB. Overall, this highlights the importance of incorporating longer‐term (beyond 1–2 months) follow‐ups and extending the duration of practice (over multiple weeks or months) in future mindfulness intervention designs. Nonetheless, there is value in interventions that support short daily practices, as traditional mindfulness programs like MBSR or MBIs require significant time and financial investments and may be unappealing or inaccessible for some organizations or individuals (Jamieson & Tuckey, 2016). The intervention used in this study required only a minimal time commitment and no financial investment, making it feasible for participants to integrate into small pockets of time. Extending the intervention duration could potentially lead to stronger effects.

This work is not without limitations. First, participants were recruited from a panel that has benefits, such as access to a diverse population but also has disadvantages related to the quality of responses (Aguinis et al., 2021; Cheung et al., 2017). Second, only two measures of mindfulness were compared to reduce the burden on participants. However, several other mindfulness measures that conceptualize and capture mindfulness in different ways are used frequently in research. It is not clear what the overlap between those measures is, and how the use of those measures affects study conclusions. Third, the second study data collection was completed during the covid‐19 wave in August–September 2020. The pandemic has caused significant mental health issues resulting in decreased well‐being (Kniffin et al., 2020; Wanberg et al., 2020) which may have affected the data. Fourth, a limitation of this study is the use of the happiness scale as a measure of SWB rather than a commonly used composite measure encompassing life satisfaction, positive affect, and negative affect (Diener et al., 1999). While the happiness scale offers a practical and parsimonious approach, it does not allow for a detailed examination of the unique contributions of mindfulness and PsyCap to the SWB subdimensions. Future research could test whether the findings replicate when employing multi‐dimensional SWB measures. While some limitations cannot be avoided in empirical work, future research can attempt to replicate these findings in different settings (different samples, using different measures, or in post‐pandemic times) to ensure their robustness or to establish boundary conditions.

The current study demonstrates how mindfulness fosters subjective and psychological well‐being through fostering psychological resources, as evidenced by the mediation effects of PsyCap. Practically, this suggests that mindfulness interventions can be designed to specifically target the development of psychological resources, such as PsyCap, to effectively enhance both subjective and psychological well‐being. While the brief online intervention had very limited effectiveness overall, it produced small but significant changes in specific mindfulness facets, such as observing and nonjudging, suggesting that short, low‐cost interventions may offer incremental benefits. Additionally, the study found that mindfulness, as measured by both the unidimensional MAAS and the multidimensional FFMQ, contributes similarly to SWB and PWB, though the FFMQ offers unique insights into the roles of individual mindfulness facets, such as describing and nonreactivity. These findings emphasize the importance of selecting appropriate mindfulness measures based on specific research or intervention goals and call for future research using additional multifaceted tools, like the Kentucky Inventory of Mindfulness Skills (Baer et al., 2004) or the Toronto Mindfulness Scale (Lau et al., 2006), to further explore mindfulness's nuanced effects.

## CONFLICT OF INTEREST STATEMENT

The author declares no conflicts of interest.

## ETHICS STATEMENT

This study received ethics approval from the University of Lethbridge Research Ethics Board. It adheres to the ethical standards of the institution and legal requirements of the study country.

## Supporting information


**Table S1.** Descriptive Statistics and Correlations of Study Variables
**Table S2.** Result From Mediation Analysis for SWB and PWB via PsyCap

## Data Availability

The data that support the findings of this study are available from the corresponding author upon reasonable request.
