# Supplementary material for: Mindfulness, subjective, and psychological well‐being: A comparative analysis of FFMQ and MAAS measures
Source: Appl Psychol Health Well Being. 2025 Mar 17;17(2):e70019. doi: 10.1111/aphw.70019 (PMC11914683; doi:10.1111/aphw.70019)
Supplement: Supplementary file 1 — Table S1. Descriptive Statistics and Correlations of Study Variables Table S2. Result From Mediation Analysis for SWB and PWB via PsyCap [file APHW-17-0-s001.docx]

# Table 1

*Descriptive Statistics and Correlations of Study Variables*

| Variable | *M* | *SD* | 1 | 2 | 3 | 4 | 5 | 6 | 7 | 8 | 9 | 10 |
| --- | --- | --- | --- | --- | --- | --- | --- | --- | --- | --- | --- | --- |
| 1. FFMQ | 3.09 | .51 |  | .43^*^ | .78^*^ | .76^*^ | .69^*^ | .72^*^ | .70^*^ | .61^*^ | .61^*^ | .57^*^ |
| 2. FFMQ-OBS | 3.24 | .65 | .46^*^ |  | .27^*^ | .05 | .28^*^ | .01 | .07 | .18 | .15 | .22^*^ |
| 3. FFMQ-DESC | 3.17 | .79 | .77^*^ | .28^*^ |  | .48^*^ | .44^*^ | .43^*^ | .47^*^ | .48^*^ | .42^*^ | .47^*^ |
| 4. FFMQ-AWA | 3.08 | .80 | .74^*^ | .08 | .45^*^ |  | .39^*^ | .56^*^ | .86^*^ | .42^*^ | .50^*^ | .45^*^ |
| 5. FFMQ-NREACT | 2.94 | .64 | .70^*^ | .25^*^ | .45^*^ | .40^*^ |  | .32^*^ | .32^*^ | .51^*^ | .48^*^ | .45^*^ |
| 6. FFMQ-NJUD | 3.02 | .83 | .73^*^ | .08 | .41^*^ | .54^*^ | .35^*^ |  | .57^*^ | .46^*^ | .50^*^ | .33^*^ |
| 7. MAAS | 3.18 | .61 | .69^*^ | .11^*^ | .44^*^ | .84^*^ | .35^*^ | .55^*^ |  | .37^*^ | .48^*^ | .41^*^ |
| 8. PsyCap | 3.78 | .56 | .61^*^ | .19^*^ | .48^*^ | .42^*^ | .50^*^ | .49^*^ | .38^*^ |  | .69^*^ | .80^*^ |
| 9. SWB | 3.34 | .90 | .60^*^ | .18^*^ | .44^*^ | .47^*^ | .44^*^ | .51^*^ | .47^*^ | .64^*^ |  | .73^*^ |
| 10. PWB | 3.98 | .75 | .54^*^ | .21^*^ | .46^*^ | .41^*^ | .40^*^ | .37^*^ | .40^*^ | .77^*^ | .69^*^ |  |
| *Note*. Correlations below the diagonal are for week-level data (N = 447). Correlations above the diagonal are for person-level data (N = 185). M = person-level means. SD = person-level standard deviations. FFMQ = Five Facet Mindfulness Questionnaire. FFMQ-OBS = observing. FFMQ-DESC = describing. FFMQ-AWA=acting with awareness. FFMQ-NJUD = nonjudgement of inner experiences. FFMQ-NREACT = nonreactivity. MAAS = Mindful Attention Awareness Scale. PsyCap = Psychological capital. SWB = subjective well-being. PWB = Psychological well-being. ^*^*p* < .05 | | | | | | | | | | | | |

# Table 2

*Result From Mediation Analysis for SWB and PWB via PsyCap*

| *Predictor* | Outcome: SWB | Outcome: PWB |
| --- | --- | --- |
| FFMQ | .07 [-.03; .19] | .17^***^ [.10; .24] |
| FFMQ-OBS | -.01 [-.04; .05] | -.02 [-.19; .16] |
| FFMQ-DESC | .00 [-.04; .05] | .01 [-.08; .10] |
| FFMQ-AWA | .04 [-.03; .13] | .11^*^ [.02; .21] |
| FFMQ-NJUD | .01 [-.01; .05] | .04 [.00; .08] |
| FFMQ-NREACT | .03 [-.02; .08] | .08^***^ [.04; .12] |
| MAAS | .10^*^ [.02; .20] | .14^***^ [.09; .21] |
| *Note*. N = 179. SWB = subjective well-being. PWB = Psychological well-being. PsyCap = psychological capital. FFMQ = Five Facet Mindfulness Questionnaire. FFMQ-OBS = observing. FFMQ-DESC = describing. FFMQ-AWA=acting with awareness. FFMQ-NJUD = nonjudgement of inner experiences. FFMQ-NREACT = nonreactivity. MAAS = Mindful Attention Awareness Questionnaire. ^*^*p* < .05, ^**^*p* < .01, ^***^*p* < .001. | | |
